# The generation of femtosecond optical vortex beams with megawatt powers directly from a fiber based Mamyshev oscillator

**DOI:** 10.1515/nanoph-2021-0537

**Published:** 2021-11-11

**Authors:** Di Lin, Yutong Feng, Zhengqi Ren, David J. Richardson

**Affiliations:** Optoelectronics Research Centre, University of Southampton, Southampton, SO17 1BJ, UK

**Keywords:** Mamyshev oscillator, mode-locked fiber laser, optical vortex beam, ultrafast pulse generation

## Abstract

Numerous approaches have been developed to generate optical vortex beams carrying orbital angular momentum (OAM) over the past decades, but the direct intracavity generation of such beams with practical output powers in the femtosecond regime still remains a challenge. Here we propose and experimentally demonstrate the efficient generation of high-peak-power femtosecond optical vortex pulses from a Mamyshev oscillator (MO) based on few-mode polarization-maintaining (PM) ytterbium-doped fibers (YDFs). By employing an appropriate intracavity transverse spatial mode selection technique, ultrafast pulses carrying OAM with selectable topological charge of *l* = ±1 are successfully generated with an average output power of ∼5.72 W at ∼24.35 MHz repetition rate, corresponding to a single pulse energy of ∼235 nJ. The chirped pulses can be compressed to ∼76 fs outside the cavity, leading to a pulse peak power of ∼2.2 MW. To the best of our knowledge, this is by far the highest pulse energy and peak power for optical vortex pulses ever generated directly from a fiber oscillator. This unprecedented level of performance should be of great interest for a variety of applications including materials processing and imaging.

## 1 Introduction

Optical vortex beams are characterized by an annular intensity profile associated with an azimuthally varying phase structure of exp(i*lφ*) (where *l* is the integer of topological charge) and that carry OAM of *lћ* per photon (where *ћ* is Planck’s constant). Such beams are subject to ever increasing attention due to their relevance and potential for widespread practical application in areas such as optical communications [1, 2], high resolution microscopy [3, 4], quantum information [5], macro-/nano-particle manipulation [6, 7] and material processing [8, 9]. Normally, such beams are generated by taking the Gaussian-profiled output beam from a laser and applying a helical phase front external to the laser cavity using a suitable spatial beam shaper – typically either a spiral phase plate [10], phase-only spatial light modulator [11], *q*-plate [12] or optical metasurface [13]. This approach works well and can be applied to both continuous-wave and pulsed lasers. However, there are often drawbacks associated with this external-cavity approach due to the power handling capabilities, modal purity, overall mode conversion loss and topological charge dispersion associated with these various beam shaping devices. These become of particular concern for the efficient generation of high average power and high peak power femtosecond optical vortex beams with their associated high intensities and broad spectral bandwidth.

The generation of optical vortex beams directly from a laser cavity generally relies on the excitation of particular eigenmodes such as higher-order Laguerre–Gaussian (LG) modes in free space cavities and Bessel modes in cavities based on circularly symmetric optical waveguides such as fibers. This therefore offers the potential for the generation of beams with high modal purity by exploiting spatial mode competition within a laser cavity. Moreover, by placing the shaping element within high gain cavities at locations in which the circulating power is much lower than the output beam power the risk of laser induced damage can be reduced. This strategy can also be used to minimize the impact of the loss of the beam shaping element on the final shaped beam power/overall laser efficiency. Many intracavity beam shaping results have been reported in recent years, both from solid state bulk [14], [15], [16], [17] and fiber oscillators [18], [19], [20]. Compared with bulk solid-state lasers, fiber lasers offer benefits in terms of higher wall-plug efficiencies, greater compactness and robustness and lower cost. Moreover, through the use of cladding-pumping, they are well suitable for high average output power operation.

Although a few techniques have been demonstrated for the generation of femtosecond optical vortex pulses from mode-locked (ML) fiber lasers, these have generally relied on the generation of traditional soliton pulses within a single mode fiber (SMF) oscillator in combination with a mode converter (e.g. a mode-selective coupler [21], two-mode long-period fiber grating [22], an acoustically induced fiber grating [23], or *q*-plate [24]) placed just before the output coupler, allowing the circulating Gaussian-shaped beam to be converted to an OAM beam in the process of being coupled out of the cavity. This underlying approach is therefore largely equivalent in practical terms to extra-cavity beam shaping and therefore it intrinsically suffers from the same drawbacks mentioned previously. The average output power of the generated femtosecond OAM beams based on such lasers is usually limited to a few tens of mW with maximum pulse energies of a few nJ and pulse durations down to a few hundred fs by the mode-locking mechanisms so far employed in these shaping experiments. However, there has been recent progress on increasing the output energy from ML fiber lasers. Using the MO concept Z. Liu et al. demonstrated that sub-50 fs pulses with a pulse energy of ∼50 nJ could be obtained directly from an SMF based ML fiber MO, representing a significant increase in achievable pulse energy relative to prior approaches [25]. Following this initial demonstration the pulse energy has been further scaled up to ∼625 nJ from an MO based on use of the fundamental mode in a step-index, few mode PM-YDF with a high quality, single mode Gaussian-shaped output beam obtained [26]. In addition, it is to be noted that few-mode step-index YDFs have also previously been successfully employed as the gain medium for directly generating/amplifying optical vortex and vector beams in various fiber laser and amplifier systems [18, 27, 28]. Here amplification in higher-order modes has been used to achieve up to 106 W of average output power and ∼180 kW of peak power for picosecond pulses [27]. The benefits of the larger mode area in terms of increased extractable pulse energy and reduced nonlinearity due to the increased mode area of the higher order modes versus the fundamental mode of the fiber were observed.

The observations above provide the incentive to explore the possibility of developing a few-mode fiber based MO with the capability to generate femtosecond optical vortex pulses with much higher pulse energy and higher average output power than the current state-of-the-art and in this paper we report the efficient generation of femtosecond optical vortex pulses with controllable topological charge of *l* = ±1 from a ring cavity MO using a few-mode PM-YDF. A *q*-plate combined with a pair of quarter-wave plates (QWPs) is employed within the cavity to convert the linearly polarized fundamental mode (LP_01_) from the first arm of the MO into a linearly polarized OAM mode that is then amplified in the second arm. A large fraction of the amplified OAM mode is coupled out of the cavity, and the residual is converted back to the LP_01_ mode which is then fed back to the first arm. The laser provides up to ∼5.72 W of average output power at a slope efficiency of ∼73% for a pulse repetition rate of ∼24.35 MHz, corresponding to a single pulse energy of ∼235 nJ. The chirped output pulses are compressed down to ∼76 fs in an external grating compressor leading to ∼2.2 MW peak powers in the final optical vortex beams.

## 2 Principles

**Figure 1:**
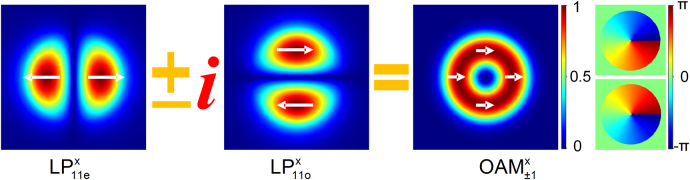
Coherent superposition of PM fiber eigenmodes ${\mathrm{LP}}_{11e}^{x}$ and ${\mathrm{LP}}_{11e}^{x}$ with ± *π*/2 phase shift results in a linearly polarized OAM mode with topological charge of *l*=±1, respectively.

In a weakly guided circular core few-mode PM fiber, the strong linear birefringence breaks the circular symmetry of the fiber eigenmodes, leading to linearly polarized fiber eigenmodes that are approximately equivalent to the familiar LP modes. We therefore designate the PM fiber eigenmodes as LP_lme_ and LP_lmo_, where l and m are the azimuthal and radial indices, respectively. The birefringence substantially lifts the difference in effective refractive index (Δ*n*_eff_ > 10^−4^) of the LP_lm_ modes between the orthogonal linear polarizations, while the LP_lme_ and LP_lmo_ modes with the same linear polarization are still nearly degenerate due to the relatively small Δ*n*_eff_ (10^−5^–10^−7^). The linearly polarized OAM modes in a PM fiber can be considered as coherent superpositions of the same linearly polarized even and odd LP_lm_ modes with ±*π*/2 phase shift, as illustrated in Figure 1. The generation of stable linearly polarized OAM modes with controllable topological charge has recently been demonstrated in passive PM fibers [29], [30], [31]. Compared with non-PM fibers, the reduced number of nearly degenerate modes in PM fibers reduces mode coupling and leads to relatively stable OAM modes. The Δ*n*_eff_ between the even and odd LP_11_ modes with the same polarization in a few-mode PM fiber with a core diameter of 25 µm and a numerical aperture (NA) of 0.065 is calculated to be ∼2 × 10^−7^ (using the COMSOL Finite Element Method software package). This leads to a mode delay of ∼1 fs/m between the LP_11e_ and LP_11o_ modes with the same linear polarization; this ensures that it should be possible to form ultrashort (sub-hundred fs) femtosecond OAM pulses in such PM fibers.

## 3 Experimental setup

**Figure 2:**
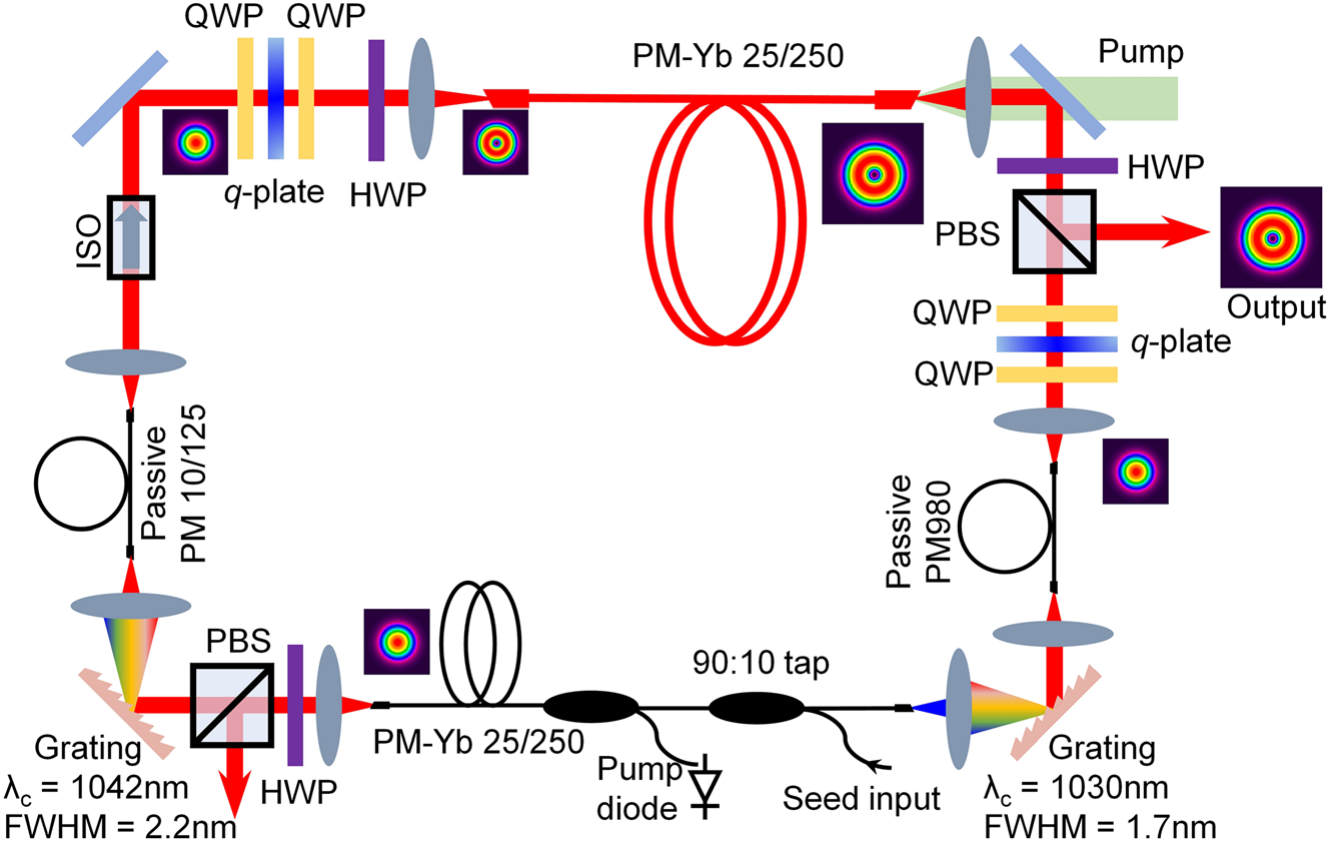
Schematic of the experimental setup. HWP: half-wave plate; ISO: isolator; PBS: polarization beam splitter.

A schematic of the experimental setup is illustrated in Figure 2, which comprises two concatenated arms. A ∼2.2 m length of PM-YDF (Nufern PLMA-YDF-25/250-VIII) is used as the gain medium in each arm. The PM-YDF has a core diameter of 25 µm with an NA of ∼0.065 and an inner cladding diameter of 250 µm. The V-number of the fiber around 1.04 µm is calculated to be ∼4.9 indicating that the fiber is capable of supporting the propagation of higher-order modes up to the LP_02_ mode. The PM-YDF in the first arm was tightly coiled with a bending diameter of ∼7 cm so that it could effectively act as an SMF by suppressing the propagation of higher-order modes by inducing high excess losses for them. The first arm thus acts as a lower-energy feedback loop for the second power scaling arm and is forward cladding-pumped with a multimode 975 nm pigtailed laser diode via a pump/signal combiner. The signal input port of the combiner has a core diameter of 10 µm with an NA of ∼0.085 and a cladding diameter of 125 µm and has a high index coating. The signal output port of the fiber combiner is a double-clad passive fiber matched to the PM-YDF which was spliced to the latter. A mode field adapter is integrated within the combiner ensuring that the fundamental LP_01_ mode is excited in the PM-YDF. A 2 × 2 PM-SMF tap with a ratio of 90:10 and fiber core diameter of 6 µm was spliced to the signal input port of the combiner, which is used for coupling both the circulated pulses from the second arm and the externally injected seed pulses needed to initiate the mode-locking. The mismatch in effective mode field diameter between the fibers of the tap and the combiner induces an extra splice loss of ∼1.2 dB. Given that the Δ*n*_eff_ between the LP_11e_ and LP_11o_ modes with the same linear *x*(*y*) – polarization is rather small (∼10^−7^–10^−6^) in this weakly guided PM-YDF, severe mode coupling between them is expected even in the case of small external perturbations. To reduce the mode coupling and to ensure the effective amplification of the first-order OAM modes within the fiber, the PM-YDF in the second arm was only loosely coiled with a large bending diameter of ∼30 cm, and any twisting of the fiber was avoided. The second arm is backward cladding pumped by a 50 W multimode 975 nm laser diode using free space coupling optics. All fiber ends of the PM-YDFs were spliced to appropriate silica endcaps with a diameter of ∼250 µm which were polished with an angle of ∼ 8° to suppress the potential for any parasitic lasing.

Two reflective ruled diffraction gratings (Thorlabs, GR25-0303) with groove densities of 300 lines/mm in combination with SMFs form the offset bandpass filters required within the MO, each offering Gaussian-shaped spectral transmission profiles – one with a center wavelength set at 1042 nm with a 2.2 nm full-width at half-maximum (FWHM), and the other at 1030 nm with a 1.7 nm FWHM passband. The large spectral separation between the two filters leads to a high modulation depth for the effective saturable absorber response they provide and this allows high energy pulses to be stabilized. The combination of the HWP and the PBS in each arm is used to provide adjustable output coupling. We minimized the output coupling ratio in the first arm to ensure the signal power seeded into the second arm is as high as possible, and the output coupling ratio of the second arm was set to ∼95% to maximize the energy of the output pulses. The combination of a pair of QWPs and a *q*-plate with appropriate rotation angles forms an OAM beam converter that converts a linearly polarized Gaussian-shaped beam into a linearly polarized OAM beam with a controllable topological charge of *l* = ±1, or vice versa [15]. The *q*-plate (Thorlabs WPV10L-1064) has a high transmittance of ∼98% around 1 µm and a laser damage threshold of 5 W/cm for continuous wave operation. Taking the theoretical mode conversion efficiency of ∼93% into account, the overall mode conversion efficiency is ∼91%. Although the *q*-plate is designed to provide half-wavelength retardance at 1064 nm, we previously employed it to convert a Gaussian-shaped beam into a radially polarized beam for picosecond pulses at a center wavelength of 1035 nm, resulting in a pronounced donut-shaped intensity profile with negligible intensity at the beam center in the far-field, which is critical for amplifying a donut-shaped mode in a fiber as any residual intensity at the beam center will be amplified significantly due to the very high gain at the center of fiber core and which is not accessible by a donut-shaped beam [27]. One beam converter is placed just before the PM-YDF of the second arm to convert the incident linearly polarized Gaussian-shaped beam emitted from the first arm into a linearly polarized OAM beam that is then coupled to the few-mode PM-YDF in order to excite the linearly polarized OAM mode within this fiber. The benefits of such a configuration are obvious. On the one hand, placing the beam converter prior to a gain fiber in which the powers are relatively low, allows the pre-shaped beam to be amplified to the desired beam with much higher output power than could be safely applied from a damage perspective directly to the beam converter external to the cavity. On the other hand, it also avoids any shaping losses and minimizes the impact of topological dispersion. Behind the output coupler of the second arm, the other OAM beam converter in combination with a short length of SMF forms a spatial mode correlation filter providing a transmission function that has a conjugate phase to the desired OAM mode and that consequently passes light in the desired linearly polarized OAM mode whilst strongly attenuating light in all other unwanted modes [32, 33]. A free-space polarization independent isolator ensures unidirectional operation (clockwise).

## 4 Experimental results

**Figure 3:**
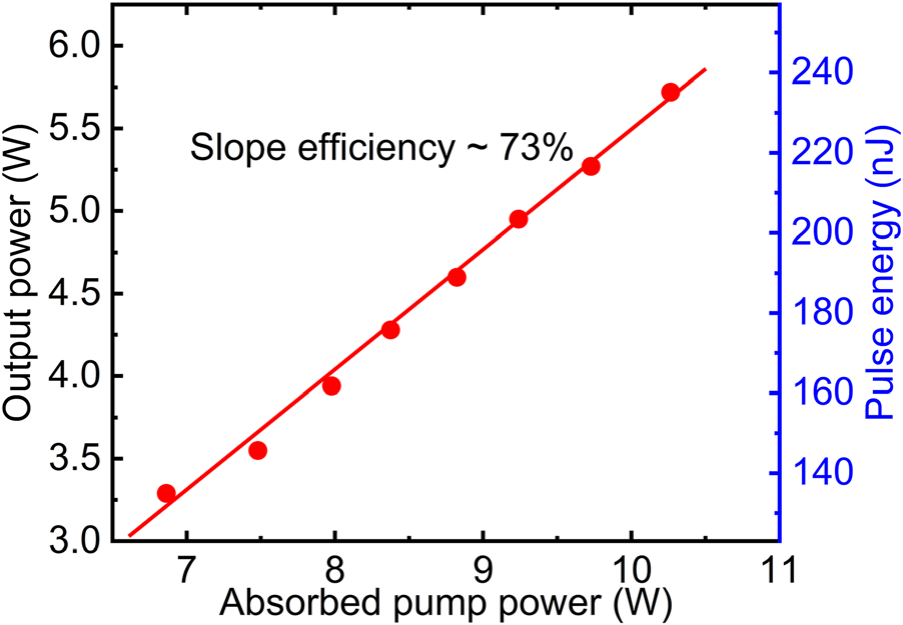
The output power and pulse energy as a function of absorbed pump power.

To initiate the mode-locking, an external seed beam emitted from an in-house-built, dispersion-managed SMF ML oscillator is employed. The output pulses are first compressed to ∼300 fs and then coupled to a 2 m length of SMF to increase the spectral bandwidth (−10 dB level) to ∼30 nm and that extends over the passbands of the two intracavity spectral filters. Chirped pulses with an energy of ∼5 pJ at a repetition rate of 23.15 MHz were launched in to the first arm through the fiber tap. The repetition rate of the seed pulse has a negligible effect on initiating mode-locking as reported in [25]. However, the pulse energy of the seed is a critical parameter in initiating mode-locking. A seed with too high a pulse energy would lead to amplification of the seed itself, while a seed with too low an energy would not enable sufficient spectral broadening to enable pulses to circulate through the full cascade of amplifiers within the cavity. After optimizing the seed/circulating signal launch conditions into the few-mode PM-YDF in the second arm and carefully manipulating the few-mode PM-YDF to reduce the inter-modal coupling, mode-locking with a donut-shaped OAM output beam can be repeatably and reliably initiated at a fundamental repetition rate of ∼24.35 MHz when the absorbed pump power is above ∼6.9 W. We observed that the mode-locking always operated within the single pulse regime above the mode-lock threshold, which is mainly due to the relatively high intracavity loss induced by the high output coupling ratio and effective intracavity spatial mode filtering. Once mode-locking was initiated we blocked the seed and gradually increased the pump power in the second arm while keeping the pump power in the first arm constant. Figure 3 shows the average output power and pulse energy of the generated ML OAM pulses as a function of the absorbed pump power in the second arm. The average output power reached a maximum of 5.72 W with a slope efficiency of ∼73% with respect to the absorbed pump power in the second arm, corresponding to a maximum pulse energy of ∼235 nJ. We monitored to ensure that an almost constant pulse energy of ∼1.5 nJ of signal was coupled to the second arm (∼1.5 dB coupling loss was measured), indicating that the second arm provided a maximum effective gain of ∼22 dB.

**Figure 4:**
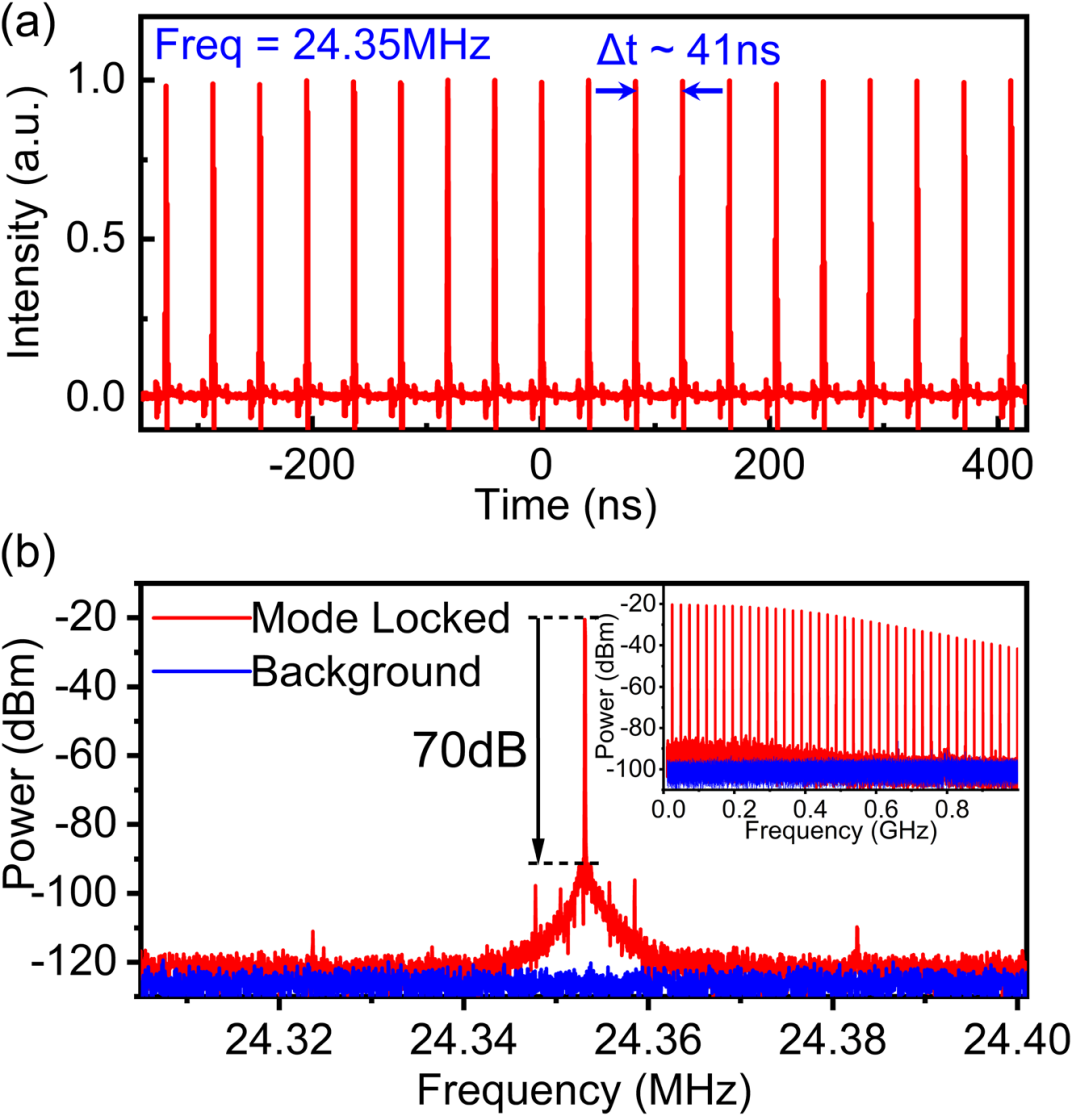
(a) Measured output pulse train. (b) Radio frequency spectrum with a RBW of 10Hz and a span of 200kHz at the fundamental frequency of 24.35MHz. Inset: RF spectrum with 1GHz span.

Figure 4(a) shows a typical measured ML pulse train with a repetition rate of ∼24.35 MHz, which is in agreement with calculations based on the cavity length and highlights the very good stability achieved. The stability of the output pulse train was further investigated using a radio frequency (RF) spectrum analyzer. As shown in Figure 4(b), the RF spectrum was recorded with a span window of 100 kHz and a 10 Hz resolution bandwidth (RBW). It shows that the fundamental beat mode has a signal-to-noise ratio of ∼70 dB, indicating highly stable mode-locking with comparable performance to more conventional ML SMF oscillators. The narrow peak also indicates relatively low amplitude fluctuation. The inset of Figure 4(b) shows the recorded RF spectrum with a span of 1 GHz and an RBW of 10 kHz, showing no sign of multiple pulsing. It is worth mentioning that the oscillator can stably operate in the mode-locking state with negligible variation in the donut-shaped intensity profile and a fixed topological charge for periods in excess of at least 2 h in a typical laboratory environment.

**Figure 5:**
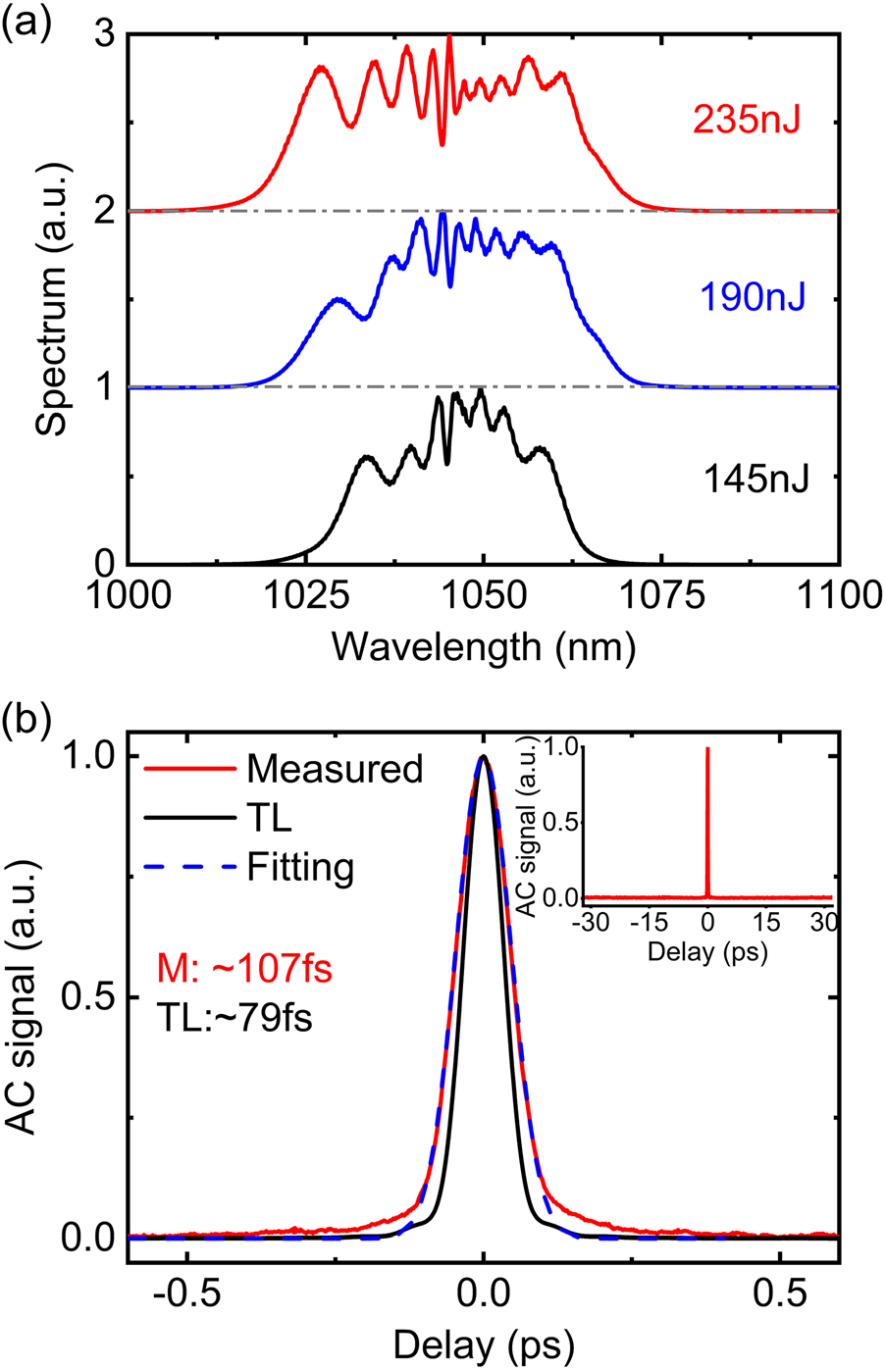
(a) Experimentally measured spectra with increasing pulse energy. (b) The measured AC trace (red curve) of the compressed pulse at the maximum pulse energy of 235nJ and the comparison with the Gaussian-fit (blue dash curve) and the calculated AC trace of the transform-limited (TL) pulse (black curve). Inset: The measured intensity AC trace with 60 ps span.

Figure 5(a) shows the output spectra directly measured after the output coupler of the second arm for different pulse energies. The spectra are as one would expect for self-phase modulation (SPM)-broadening of almost unchirped Gaussian pulses [34]. The bandwidth at the −10 dB level increases from ∼40 nm to ∼60 nm (ranging from 1010 nm to 1070 nm) as the pulse energy increases from 145 nJ to 235 nJ. The spectra exhibit asymmetric profiles due mainly to the self-steepening effect and pulse distortions due to third-order dispersion. The intensity autocorrelation (AC) duration of the chirped output pulses was measured to be ∼2.8 ps. The output pulses were successfully compressed using a pair of transmission gratings (1000 lines/mm) with a compression efficiency of ∼72%, resulting in a maximum compressed pulse energy of ∼169 nJ. We observed that the AC duration of the compressed pulses decreased with an increase in the pulse energy. The duration of the AC trace of the compressed pulse was measured to ∼107 fs (FWHM) at the maximum pulse energy as shown in the red curve in Figure 5(b), which is reasonably well fitted with the calculated AC trace for a Gaussian-shaped pulse as shown in the blue-dash curves. The calculated TL pulse has an AC duration of ∼79 fs as shown by the black curve, which is ∼1.35 times shorter than the measured pulse. Assuming a Gaussian pulse shape, the temporal duration of the compressed pulses in the OAM beam is estimated to ∼76 fs with a peak power of ∼2.2 MW. The deviation between the measured and the TL AC duration can be attributed to higher order phase induced by nonlinear effects and the grating compressor itself. The clean and single peak AC trace measured with a long range (64 ps) delay window is shown in the inset of Figure 5 excludes the existence of any broad pedestal underneath the signal and potential multiple pulses.

**Figure 6:**
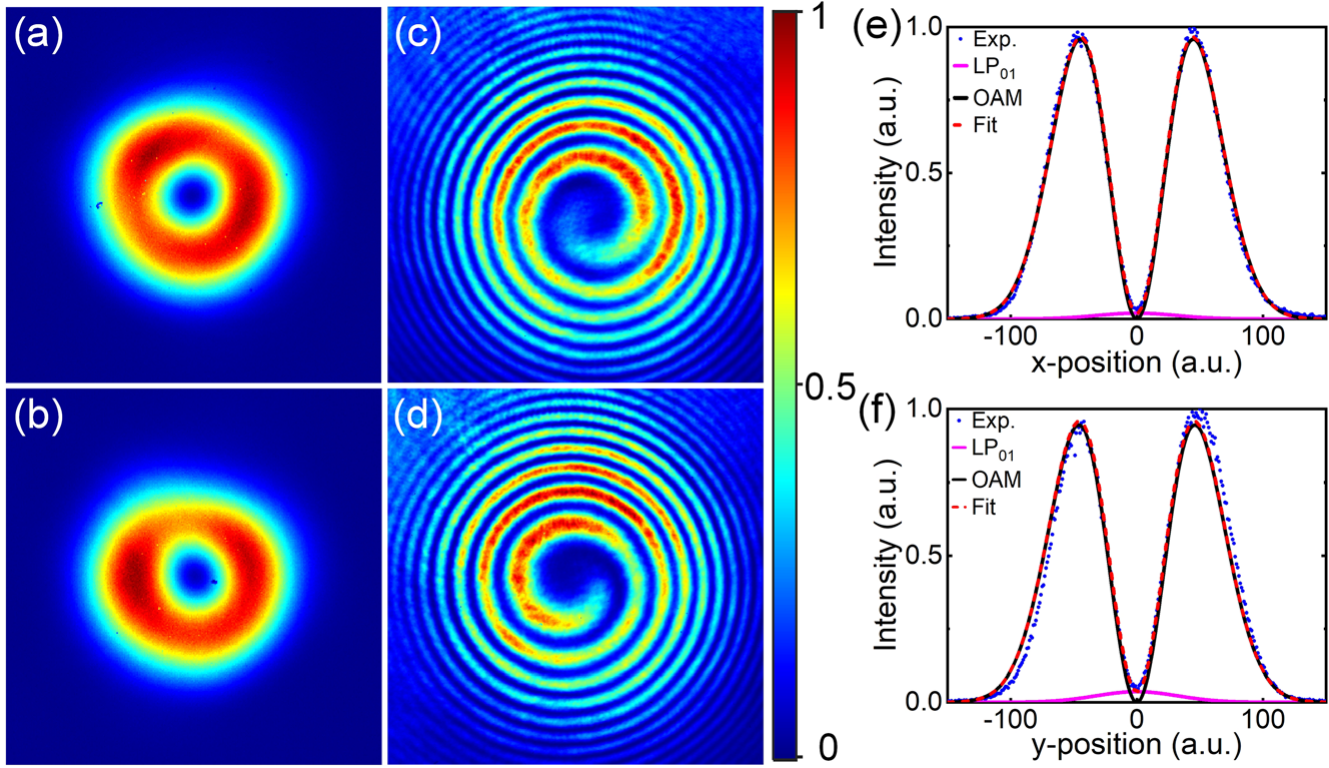
Measured far-field intensity distributions for the generated OAM beams with topological charge of (a) *l*=1 and (b) *l*=−1; (c) and (d) interference patterns of the corresponding beams; intensity distribution across the beam center along (e) horizontal and (d) vertical axis corresponding to the beams in (a) and (b), respectively. The blue dot curves are the measured intensity profiles, the solid dark curves and solid pink curves represent the theoretical intensity profiles of the OAM mode (|*l*|=1) and LP_01_ modes, respectively; the short dash red curves represent the incoherent superposition of the intensity profiles of the LP_01_ and OAM modes.

Figure 6(a) and (b) show typical spatial intensity distributions for the output OAM beams with opposite handedness of helical phase front measured at the maximum output power, respectively. Both beams show a pronounced donut-shaped pattern. The slight deviation from an ideal donut-shape can be attributed to a slight power imbalance between the orthogonal LP_11e_ and LP_11o_ modes resulting from residual inter-modal coupling. The OAM mode purity was measured to ∼93% with the mode decomposition method as described in [33]. Figure 6(c) and (d) show the measured interference patterns of the corresponding output beams presented in Figure 6(a) and (b), respectively. The spiral fringes with opposite rotation directions confirm the opposite topological charge of the helical phase front for the generated OAM beams. The change of topological charge of OAM beams was simply achieved by rotating the fast axes of the QWPs in both beam converters by 90° (which required that the mode-locking be re-initiated). It is worth mentioning that the output OAM beam can sometimes exhibit a degree of beam distortion as a result of susceptibility to mode coupling between the orthogonal LP_11e_ and LP_11o_ modes due to perturbations, (primarily bend and thermally induced), during the propagation and amplification through the fiber due to the relatively small Δ*n*_eff_. However, we found that it was possible to optimise the quality (e.g. cylindrical symmetry) of the donut intensity profile of the output beam by applying a controlled level of stress to the PM-YDF in the horizontal plane in order to adjust the relative weighting and phase between these two orthogonal modes. This had no impact at all on the handedness of the helical phase front. The blue dots in Figure 6(e) and (f) show the measured one-dimensional intensity distributions across the center of the beams shown in Figure 6(a) and (b) along the *x*-position and *y*-position, respectively. It is clear that there is some low level residual intensity at the center of the beam which we believe to be due to amplified spontaneous emission (ASE) within the fundamental LP_01_ mode. The measured intensity profiles can be fitted well with incoherent intensity superposition of the fundamental Gaussian mode (LG_00_) and the first-order (|*l*| = 1) OAM mode (namely the first higher-order Laguerre–Gaussian mode (LG_01_) in free-space, represented by the red short-dash curves). The power weighting of the OAM mode is calculated to > ∼98.6%. We observed that any further increase in the absorbed pump power beyond ∼10.8 W would significantly increase the power in the LP_01_ mode and hence result in unstable mode-locking and that eventually mode-locking would be suddenly lost once the threshold for parasitic lasing on the fundamental LP_01_ mode was exceeded. We believe this to be the main reason we were unable to further scale the pulse energy of our OAM pulses.

## 5 Conclusions

In summary, we have proposed and demonstrated a novel approach to efficiently generate high energy femtosecond OAM pulses with controllable topological charges of *l* = ±1 directly from a ring-cavity ML fiber MO based on commercially available few-mode PM-YDFs. The generated optical vortex pulses are formed from the coherent superpositions of the two linearly polarized LP_11o_ and LP_11e_ fiber eigenmodes with ±*π*/2 relative phase shift. The experimental results indicate that the relative phase between the orthogonal LP modes can be maintained even in the presence of very strong nonlinearly induced spectral broadening, resulting in OAM pulses with high peak power and high modal purity. The MO reaches an average output power of ∼5.72 W at a repetition rate of 24.35 MHz, corresponding to a single pulse energy of ∼235 nJ. The generated OAM pulses have a spectra bandwidth of ∼60 nm at the −10 dB level and can be dechirped external to the cavity to achieve pulses with an AC duration of ∼107 fs. Assuming a Gaussian-shaped pulse, the temporal width of the OAM pulses is estimated to ∼76 fs, leading to an estimated peak power of ∼2.2 MW. The maximum ML output power is mainly limited by the build-up of ASE in the fundamental LP_01_ mode and ultimately parasitic lasing. This might be improved by using more sophisticated bulk end-caps to better suppress parasitic lasing and/or by more advanced fibers which have a ring-shaped Yb-doping profile. The demonstrated ultrafast OAM pulse generation and power-handling capabilities of our approach opens a new pathway to the generation of high peak power, high average output power ultrashort OAM pulses from simple and compact fiber oscillators. The laser may prove useful for a wide range of applications including laser-based material processing and high-resolution medical imaging.

## Supplementary Material

Supplementary Material
